# Whole Breast Irradiation with Halcyon™ 2.0: Workflow and Efficiency of Field-in-Field Treatment with Dynamic Beam Flattening Technique and kV Cone Beam Computed Tomography

**DOI:** 10.7759/cureus.3510

**Published:** 2018-10-29

**Authors:** Chris Kennedy, Gary Freedman, Neil Taunk, Ryan Scheuermann, Lei Dong, James M Metz, Taoran Li

**Affiliations:** 1 Department of Radiation Oncology, Perelman School of Medicine, University of Pennsylvania, Philadelphia, USA

**Keywords:** radiation therapy, whole breast irradiation, halcyon, flattening filter free, dynamic beam flattening

## Abstract

Whole breast irradiation accounts for a substantial fraction of patients treated in Radiation Oncology clinics. The recently introduced Halcyon™ platform provided a high-throughput, simplified workflow. The 2.0 version introduced new features such as the dynamic beam flattening (DBF) technique that uses the upper layer of the multi-leaf collimator (MLC) to create a flat beam profile at depth and an improved kV cone beam computed tomography (kV CBCT). In this case report, we described our experience in whole breast irradiation with Halcyon 2.0 new features. The patient was simulated in the supine position with the same immobilization technique used on C-arm linacs and an additional contralateral elbow position measurement to ensure clearance. The treatment planning process using DBF and field-in-field technique was similar to the traditional flattened beam planning and did not require additional training. Dosimetric analysis showed satisfactory dose-volume histogram (DVH) parameters that met all the planning objectives, with maximal dose at 107% and V105% at 3.6% of the breast volume. Daily image-guided radiation therapy (IGRT) using improved CBCT showed excellent soft tissue contrasts and sufficient field of view. The average imaging and treatment time was nine minutes, and the average in-room time was 16.2 minutes. These treatment times were substantially higher than those for breast treatments on our Halcyon platform using an irregular surface compensator technique. The use of DBF contributed to the majority of treatment time increase due to the motion of the upper layer of the MLC to create a flat beam profile. The total treatment time using DBF might be too long for patients with deep inhalation breast hold (DIBH) and can be drastically reduced using an irregular surface compensator technique, also known as the electronic tissue compensation (ECOMP) technique, instead of the DBF-enabled field-in-field technique.

## Introduction

Breast cancer is the most prevalent non-skin cancer among women in the United States [1-2]. Whole breast radiation therapy after lumpectomy is used to control early-stage breast cancer as an alternative to mastectomy [3]. Currently, the most widely used treatment planning technique for adjuvant radiation in the United States is 3D field-in-field treatment plan using tangential treatment fields on a C-arm linac [4-6]. This 3D field-in-field method is simple, efficient, and relatively effective in producing a uniform dose distribution within the breast.

Halcyon™ (Varian Medical Systems, Palo Alto, CA) treatment delivery system was a newly introduced design that features a high-throughput, simplified workflow. The Halcyon features a closed design with all linac components mounted on a ring structure and a bore with a 100-cm-diameter opening. The key differences between the Halcyon and the regular C-arm linacs include the omission of the light field and optical distance indicator (ODI), the requirement of daily image guidance, and the only energy choice of 6MV flattening filter-free (FFF) beams.

Traditionally, breast irradiation on a C-arm linac is usually performed with 6 MV alone or 6 MV mixed with higher energy without daily imaging: patient set up usually relies on field light agreement with skin marks and source-to-surface distance confirmation (SSD) check. In the treatment planning stage, dosimetry is also accustomed to creating field-in-field treatment plans based on the flat beam profiles. With Halcyon, these options are no longer available, representing a change in the workflow and planning strategies. The native energy from Halcyon is 6FFF that has a non-flat beam profile. This non-flat profile makes it difficult to create simple field-in-field treatment plans using the existing experiences within the dosimetry team [7].

On the first release of Halcyon 1.0 platform because of the non-flat beam profile, our institution chose to use an irregular surface compensator for breast planning on Halcyon. The irregular surface compensator technique, also known as the electronic tissue compensation (ECOMP) technique, utilizes a continuously varying fluence map to compensate the tissue depth change in tangential beams to achieve a uniform dose at a certain depth. The fluence map is then converted to dynamic multi-leaf collimator (MLC) motion for beam delivery. The ECOMP technique has been shown to have superior dose homogeneity compared to the traditional intensity-modulated radiation therapy (IMRT) and tomotherapy techniques [8]. However, ECOMP planning requires a lot of manual fluence editing and relies heavily on the planner’s experience. Therefore, it requires both additional training upfront and longer planning time. In addition, because of its dynamic MLC delivery nature, ECOMP plans will require quality assurance (QA) of IMRT to be done as per institutional standards, which requires an additional physics resource.

Halcyon 2.0 now includes an improved kV CBCT with a fast acquisition, lower dose, and an iterative reconstruction [9] and a unique dynamic beam flattening (DBF) MLC sequence that creates a flat beam profile at depth using an MLC. Because Halcyon uses two-layer stacked MLCs, this new DBF MLC sequence automatically flattens the field using the upper layer of the MLC by a fixed sequence that does not change with the patient and use the lower layer of the MLC to provide beam shaping and aperture definition. A comparison of the beam profiles with and without the DBF sequence has been detailed and illustrated in the subsequent sections. 

This case report describes our experience with administrating breast irradiation with Halcyon 2.0 utilizing both improved kV CBCT capability and DBF with the traditional field-in-field treatment planning technique.

## Case presentation

Patient workup and prescription dose

**Figure 1:**
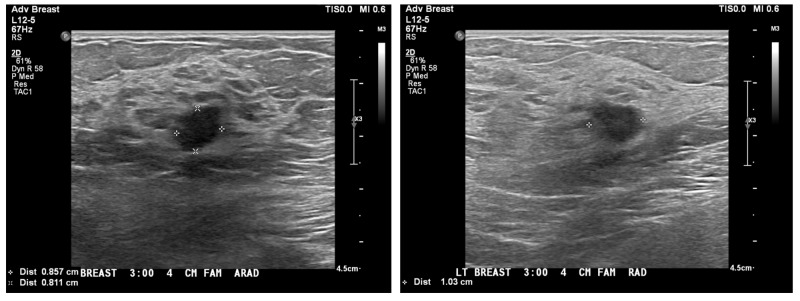
Ultrasound images showing the size of the the mass in the left breast

The patient presented with an abnormal screening mammogram. An ultrasound confirmed a 9 x 8 x 10-mm mass, as shown in Figure 1. She was diagnosed with a left-sided breast cancer involving the upper outer quadrant. Final pathology after lumpectomy revealed a 1-cm infiltrating ductal carcinoma/AJCC (The Americal Joint Committee on Cancer) Stage IA, pT1b pN0(i+) cM0, grade 2, ER+, PR+, HER2/neu negative. There were two negative sentinel lymph nodes. The Oncotype Dx Risk Score was 13. No chemotherapy was needed, and the patient was advised to receive adjuvant breast radiation to complete breast-conserving therapy.

The patient received left whole breast radiation without regional node irradiation on the Halcyon platform. Treatment prescription was 266 cGy x 16 fx = 4256 cGy using tangent fields.

CT simulation and patient positioning

The patient was oriented in the head-first supine position on the Qfix angle board with an arm shuttle (Qfix, Avondale, PA). Both arms were extended, with hands grasping the arm shuttle’s poles behind the patient. The breast board angle was set to 10 degrees to level the sternum.

Prior to simulation, the physician placed wires to delineate the breast volume, surgical scar, and the longitudinal extent of the treatment volume. Field markers were placed at the presumed superior, inferior, lateral, and medial edges of a standard tangential beam using the clinical breast volume as a reference. CT spots were placed on the mid-sternal line prior to the scan to define the setup isocenter approximately midway between the superior and inferior wires. The CT simulation scan extended from the chin through lungs. The reconstructed slice thickness was 3 mm, and the reconstruction field of view was 65 cm. The patient was marked at the locations of the field borders mentioned above.

**Figure 2:**
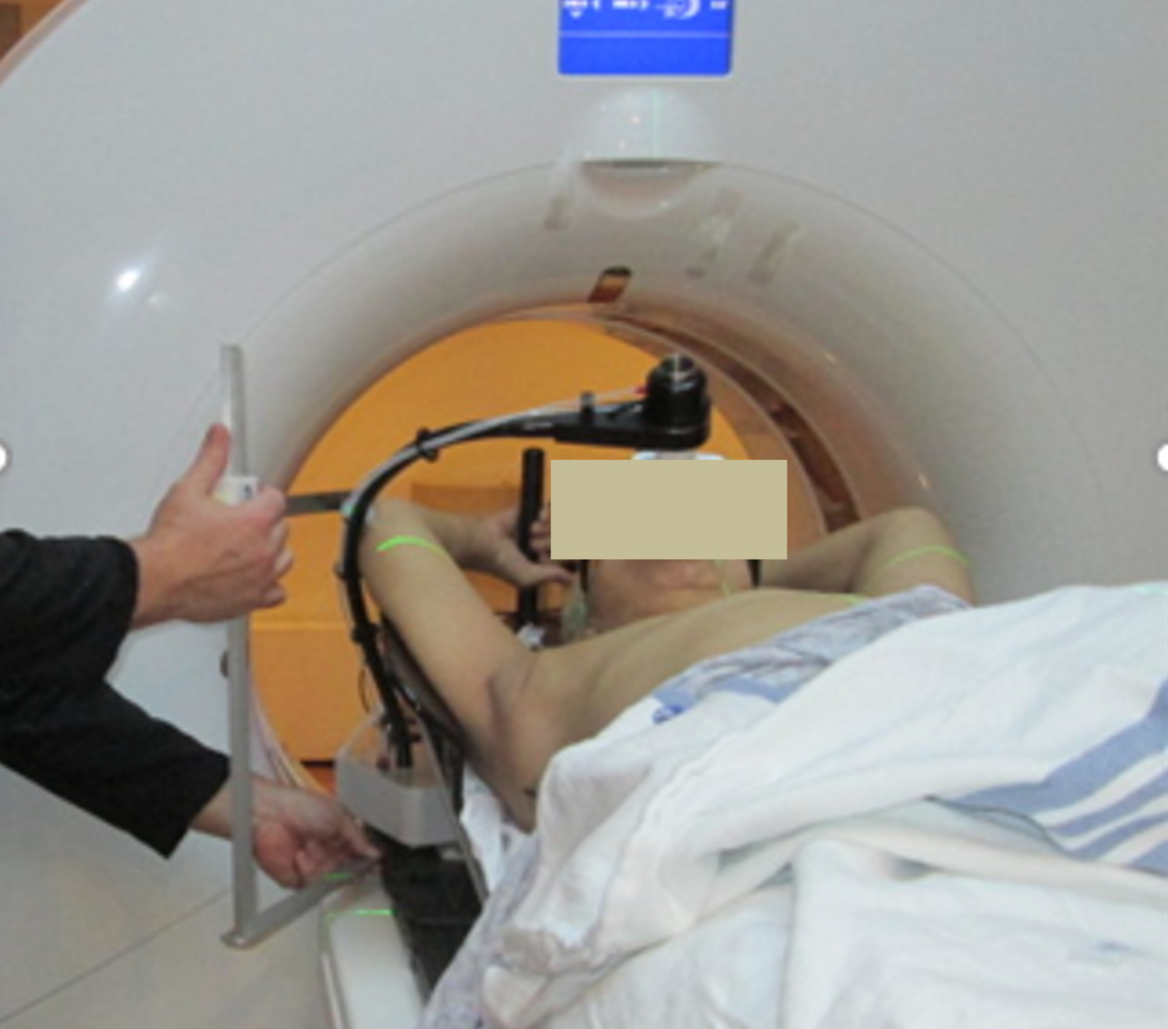
Measurement of the contralateral elbow position performed at the time of simulation to ensure clearance with the Halcyon bore (picture showing a different case where DIBH device was used) DIBH: deep inhalation breast hold

Measurements of the contralateral elbow position relative to the CT table and patient midline were made by the simulation therapists to assess potential collision with the Halcyon bore (Figure 2). Patient positioning, immobilization, and image reconstruction settings were all consistent with the institutional standards for this type of treatment with the exception of the measurements to assess potential Halcyon bore collision. If no sufficient clearance was found, the patient would be sent to a regular C-arm linac for better elbow clearance. 

Daily imaging selection

The kV-CBCT images were selected for daily localization. The imaging instructions for this case were to match the chest wall and ensure that all breast tissues were included in the BREAST_PTV structure. The planning target volume (PTV) structure was created in accordance with the contouring guidelines recommended by the Radiation Therapy Oncology Group (RTOG). This includes creating a whole breast clinical target volume, a uniform expansion to create a PTV, and a PTV evaluation structure cropped back from the ribs and 5 mm under the skin to allow for buildup. Having daily volumetric images provided our clinician with additional information and confidence of the patient setup that previous orthogonal and portal images on C-arm linac often lacked.

Treatment planning process using DBF

**Figure 3:**
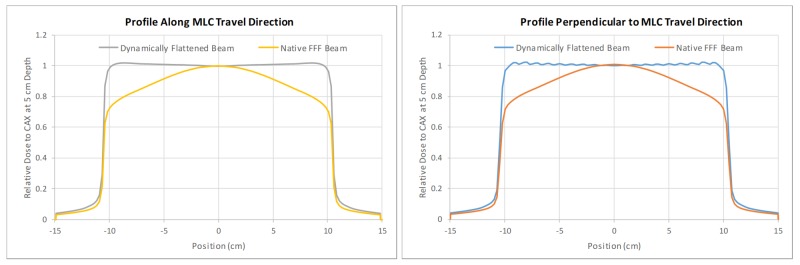
Comparison of beam profiles in both X and Y directions between Halcyon’s native 6FFF beam and DBF sequence using upper layer MLC motion; ripples in the flattened beam profile perpendicular to the MLC travel caused by the tongue-and-groove effect DBF: dynamic beam flattening; MLC: multi-leaf collimator

DBF is a new feature introduced in Halcyon 2.0 that utilizes the dynamic motion of the upper layer of the MLC for a flat beam profile at depth, although the native beam profile is non-flat. Essentially, the upper (proximal to the source) layer of the MLC creates a non-uniform fluence that is lower in the center and higher in the peripheral area of the field to compensate for the radial intensity fall-off from the native non-flat beam. DBF does not impact the maximal field size achievable with Halcyon, which is 28 x 28 cm. Comparison of the beam profiles with and without the DBF sequence is shown in Figure 3. It should be noted that the profiles in this figure are normalized to the central axis. The percentage increase in the monitor unit (MU) for delivering the same dose at 5-cm depth due to the DBF sequence was 22% for 6 x 6 cm, 38% for 10 x 10 cm, 143% for 20 x 20 cm, and 169% for 28 x 28 cm fields. This means DBF-enabled beams will generally require a substantially more MU to deliver the same dose. 

The initial tangent field placement was performed by the physician in the treatment planning system using a TrueBeam machine model to set the gantry, collimator, and jaw positions defining the treatment extent. This step was to follow the institutional standards for the field definition process. The dosimetrist then used these fields to define the Halcyon fields with the same parameters. The TrueBeam fields were calculated to generate a structure from the 50% isodose volume. This structure was used to define an initial aperture for the open tangent Halcyon MLC fields. A field-in-field approach using the DFB-enabled static fields was then performed to achieve the following institutional dose objectives listed in Table 1. It should be noted that it is not necessary to use the TrueBeam fields as guidance as a user can perform the same field shaping in Halcyon using DBF and MLC. 

**Table 1:** Treatment planning objectives used by dosimetrist to generate the treatment plan for this patient. The lower number in the fifth column indicates higher priority.

Target and Critical Normal Tissue Constraints				
Structure Name	DVH Objective	Evaluator	Variation Acceptable	Priority
PTVeval_BREAST	D95%[Gy]	≥ 40.4	≥ 38.3	1
PTVeval_BREAST	V90%[%]	≥​​​​​​​ 99	≥​​​​​​​ 98	2
PTVeval_BREAST	V105%[%]	≤​​​​​​​ 10	≤ ​​​​​​​ 15	2
PTVeval_BREAST	Max[%]	≤ ​​​​​​​ 107	≤ ​​​​​​​ 110	1
BREAST_CONTRA	D5%[Gy]	≤ ​​​​​​​ 2	≤ ​​​​​​​ 3	3
LUNG_IPSI	V20Gy[%]	≤ ​​​​​​​ 15	≤ ​​​​​​​ 20	2
LUNG_IPSI	V5Gy[%]	≤ ​​​​​​​ 50	≤ ​​​​​​​ 55	2
LUNG_CONTRA	V5Gy[%]	≤ ​​​​​​​ 10	≤ ​​​​​​​ 15	3
HEART	V20Gy[%]	≤ ​​​​​​​ 2	≤ ​​​​​​​ 5	2
HEART	Mean[Gy]	≤ ​​​​​​​ 3	≤ ​​​​​​​ 4	2

**Figure 4:**
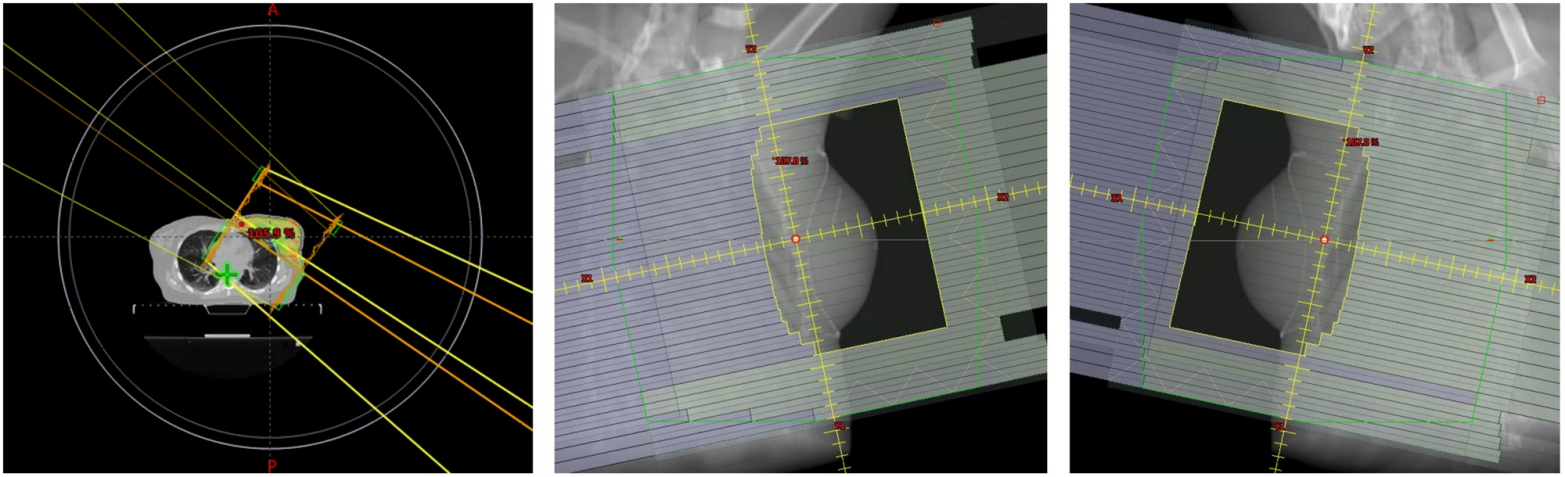
Left: Patient positioning relative to the Halcyon bore (outer circle) and beam arrangement; middle and right: medial and lateral beam aperture

In Figure 4, the image on the left-hand side represents the patient positioning and extent relative to the bore. The solid circle represents the bore size, and the inner smaller circle represents 2.5 cm away from the actual bore location. In Figure 4, the images in the middle and the right-hand side show the open apertures of the medial and lateral beams, respectively.

**Figure 5:**
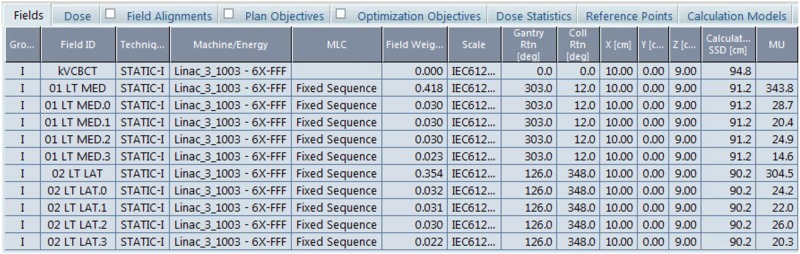
Field settings for the treatment plan

The resulting field settings for the treatment plan are shown in Figure 5. The patient separation at mid-tangent measured 23.0 cm on the medial field border. Notice the MUs are considerably higher than the typical 6X-based field-in-field breast treatment plan using tangential beams (150-200 MU per beam) for 266cGy per fraction. 

**Figure 6:**
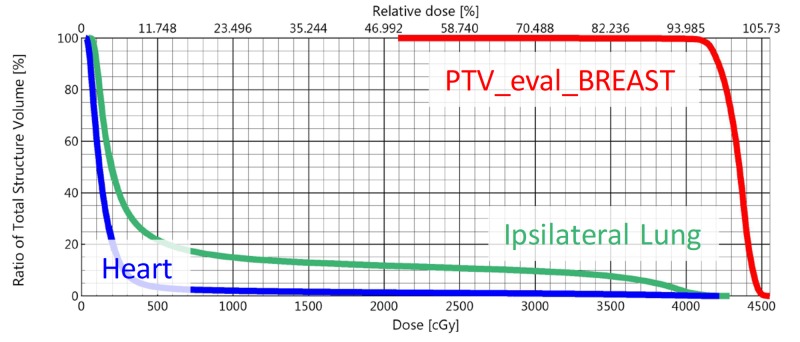
DVH of the treatment plan showing the key structures DVH: dose-volume histogram

The dose-volume histogram (DVH) for the combined plan sum is shown in Figure 6. The maximum dose for the plan was 107.0% of prescription, and 3.6% of the planning volume received 105% of the prescription dose. All planning objectives on organs at risk were met.

The dosimetrist felt that the planning efforts required for this case were comparable to a similar C-arm linac plan. Because the DBF sequence is transparent to the planner, the planner did not feel that any additional training was needed. The lack of additional training required for specialized planning techniques for some dosimetrists transitioning from traditional C-arm linac planning is an advantage of the field-in-field planning method using DBF.

Treatment delivery summary

**Figure 7:**
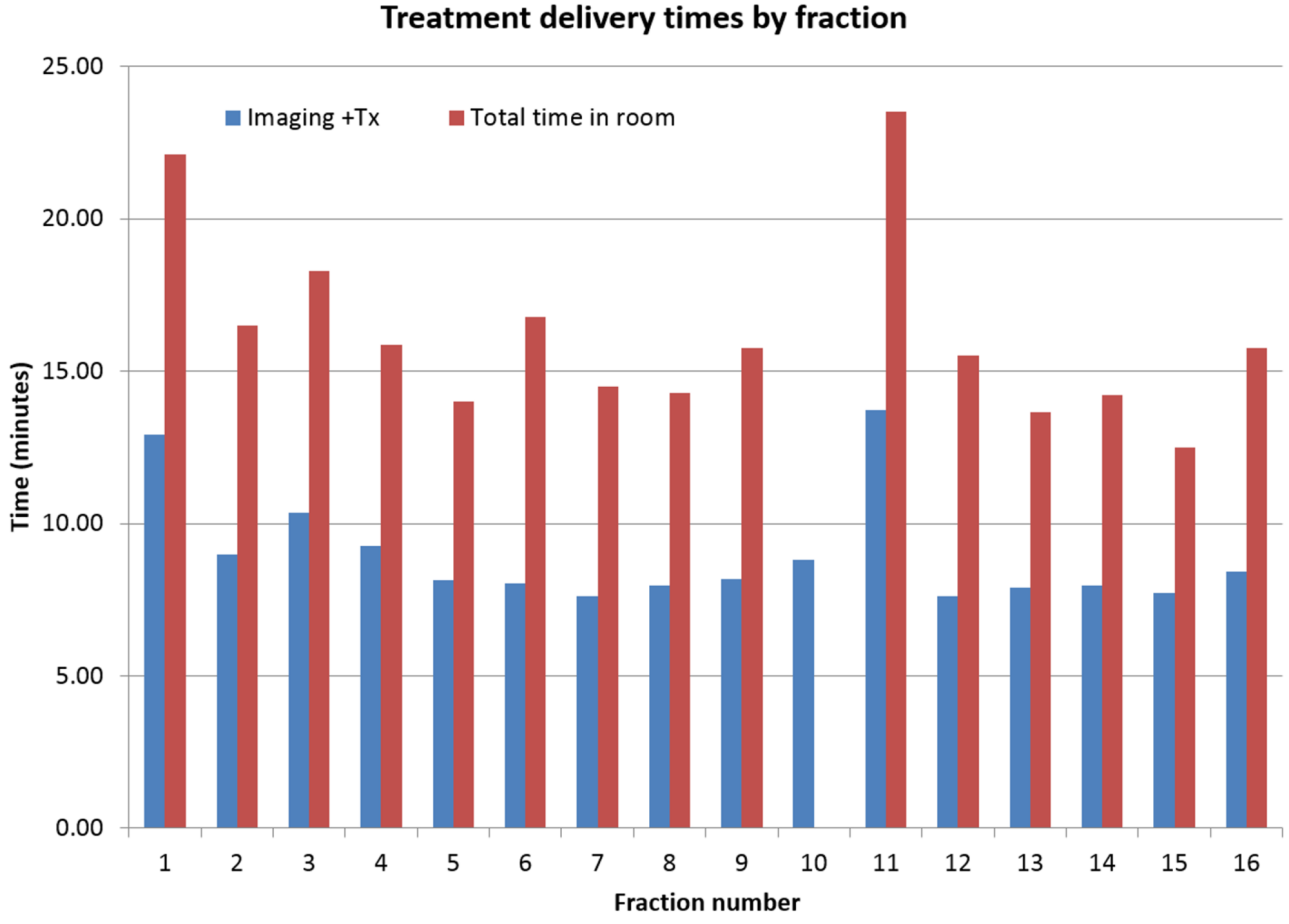
Treatment delivery times by fraction

The patient appointment length was 15 minutes per fraction except fractions 1 and 11 for which the appointment length was 30 minutes. The additional time for these fractions is due to the necessity of physician approval of imaging prior to treatment. The daily combined imaging and treatment delivery time and total treatment room time are shown in Figure 7. The average time between the start of imaging and completion of treatment was nine minutes, and the average total time in the treatment room was 16.2 minutes. The total time in the treatment room for fraction 10 was not included in the analysis due to a mechanical problem with the couch.

**Figure 8:**
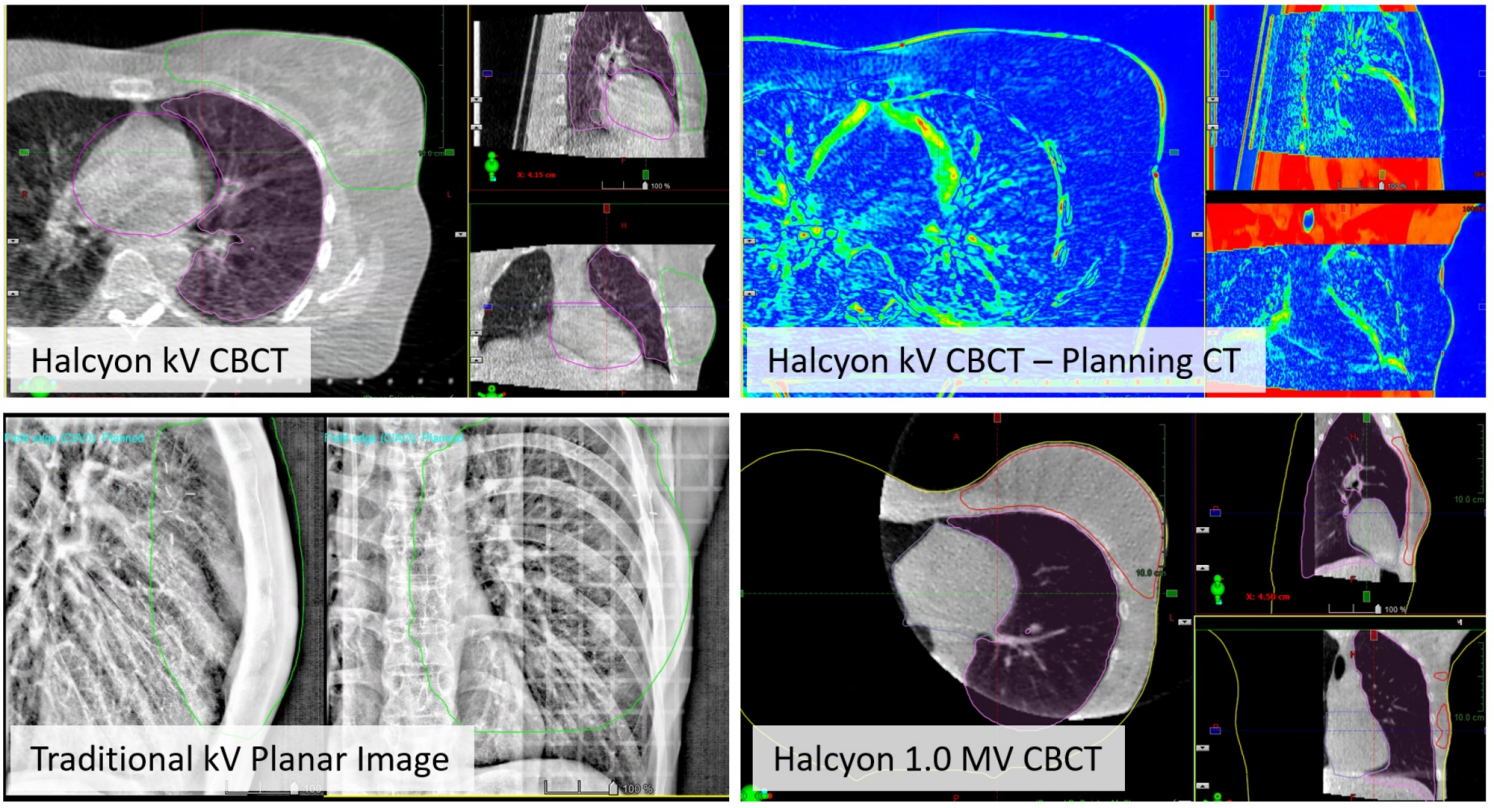
Top row: Online IGRT with improved kV CBCT of the tangent fields using the breast CBCT preset. Overlays of the heart, ipsilateral lung, and BREAST_PTV structures from the planning CT allow assessment of alignment quality. The image on the right shows the difference between planning CT and online CBCT. Bottom row: Examples of breast localization with traditional kV planar image pair and Halcyon 1.0 MV CBCT IGRT: image-guided radiation therapy; CBCT: cone beam computed tomography; PTV: planning target volume

For the tangent fields, the kV-CBCT image quality was excellent for alignment to the chest wall, and verification that the breast, heart, and the ipsilateral lung structures were in good agreement with the planning CT. One example of the daily CBCT to planning CT registration result is shown in the top row of Figure 8. Excellent soft tissue contrast within the breast tissue was observed. For reference, the bottom row in Figure 8 shows breast treatment localization using the traditional kV planar image (left) and Halcyon 1.0 MV CBCT (right). Notice the lack of 3D information from the planar image as well as the lower soft tissue contrast and limited field of view in the MV CBCT. 

No difference in physician-reported Common Terminology Criteria for Adverse Events (CTCAE) skin reaction was noted relative to the institutional standard treatment of field-in-field delivery on TrueBeam Linac with 6MV or 6MV mixed with higher energies.

For daily positioning, an external sagittal laser was found to be beneficial for patient straightening due to the limited extent of the internal Halcyon sagittal laser. Re-imaging was not necessary for any fraction. The maximum difference between the auto-match defined shifts and the manually defined shifts for any treatment fraction was 3 mm. 

## Discussion

The Halcyon 2.0 platform provided simplified workflow, fast delivery, and improved image-guided radiation therapy (IGRT) capabilities for breast treatment without the need to address possible collision during treatment delivery. The newly introduced DBF technique enables dosimetrists to use the same field-in-field technique on 6MV FFF beams without additional training, at the cost of substantially increased MU and delivery time. 

**Figure 9:**
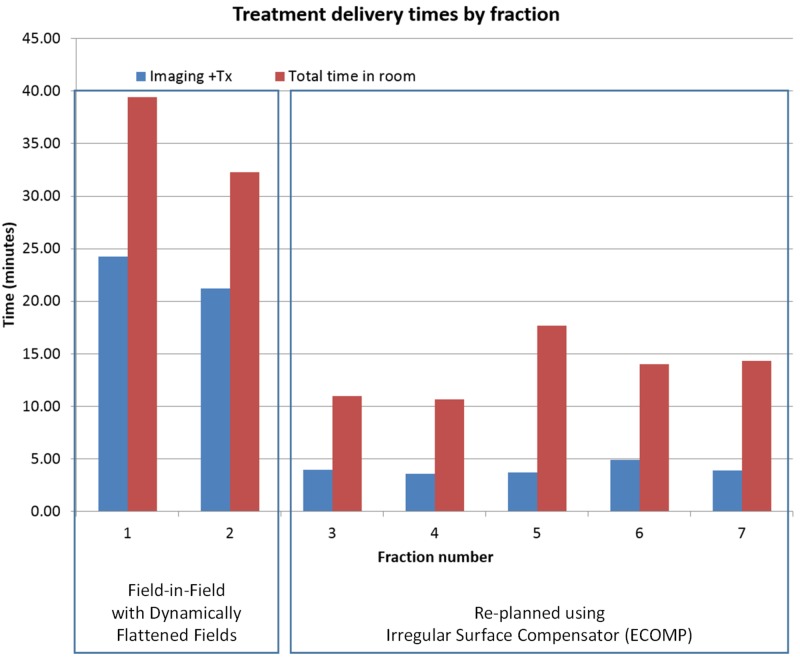
Treatment delivery times by fraction for a left-sided breast cancer patient treated with DIBH; planning techniques switched from DBF-enabled field-in-field (fractions 1 and 2) to irregular surface compensator (fraction 3 and onward) on the same Halcyon treatment unit DIBH: deep inhalation breast hold; DBF: dynamic beam flattening

The average treatment time of nine minutes for this case utilizing DBF is significantly longer than the breast cases planned with irregular surface compensators on the Halcyon treatment platform, which typically take approximately 3-4 minutes for delivery including the IGRT process based on our experience. This additional delivery time is especially problematic for deep inspiration breath hold (DIBH) patients due to the additional necessary breath holds. Multiple breath holds performed with the voluntary breath hold device (e.g. SDX) are required for each field. To illustrate the problem, treatment data for another left-sided breast case treated with DIBH and planned with DBF are shown in Figure 9. The patient was re-planned with irregular surface compensator fields after the second fraction due to the extended delivery time. The total treatment time for the DBF plan treated under DIBH averaged 23 minutes per fraction. The average treatment time was reduced to 5.5 minutes for the re-plan with an irregular surface compensator on the same Halcyon treatment unit. The treatment times include the IGRT process.

We have decided to discontinue the use of DBF-enabled fields for DIBH patients on Halcyon due to the extended treatment time.

There might be concerns regarding the increase in leakage and transmission radiation through a single-layer MLC when using DBF, especially considering the increased MU. The single-layer MLC of Halcyon has a leakage of 0.4% to 0.7% compared to ~1.5% in TrueBeam linacs. Therefore, even though only a single layer is used in the DBF-enabled fields, the leakage/transmission radiation through the proximal leaf is still less or comparable to the TrueBeam plans with the field-in-field or ECOMP techniques.

Another challenging scenario for treating breast cancer patients with Halcyon platform is when nodal irradiation is involved, e.g. supraclavicular, IMN, and or axillary nodes. We have been exploring planning techniques for these types of cases using a combination of the irregular surface compensator, DBF, and other newly introduced features with Halcyon 2.0. We hope to report a successful case soon.

## Conclusions

This report details our experience performing whole breast irradiation on a Halcyon 2.0 linac utilizing improved kV-CBCT image guidance and DBF feature to enable a similar planning process as the regular field-in-field technique. The overall simulation and planning process was very similar to breast planning on C-arm linacs. Although DBF removed additional required training to the dosimetry team to plan a breast case on a Halcyon platform using 6MV FFF energy, delivery efficiency was reduced using a DBF MLC sequence. The total treatment time can be drastically reduced using irregular surface compensator technique as opposed to the DBF-enabled field-in-field technique.
